# Circuit Organization Underlying Optic Flow Processing in Zebrafish

**DOI:** 10.3389/fncir.2021.709048

**Published:** 2021-07-21

**Authors:** Koji Matsuda, Fumi Kubo

**Affiliations:** ^1^Center for Frontier Research, National Institute of Genetics, Mishima, Japan; ^2^Department of Genetics, SOKENDAI (The Graduate University for Advanced Studies), Mishima, Japan

**Keywords:** optic flow, direction selective cells, pretectum, zebrafish, optokinetic response, optomotor response, cerebellum

## Abstract

Animals’ self-motion generates a drifting movement of the visual scene in the entire field of view called optic flow. Animals use the sensation of optic flow to estimate their own movements and accordingly adjust their body posture and position and stabilize the direction of gaze. In zebrafish and other vertebrates, optic flow typically drives the optokinetic response (OKR) and optomotor response (OMR). Recent functional imaging studies in larval zebrafish have identified the pretectum as a primary center for optic flow processing. In contrast to the view that the pretectum acts as a relay station of direction-selective retinal inputs, pretectal neurons respond to much more complex visual features relevant to behavior, such as spatially and temporally integrated optic flow information. Furthermore, optic flow signals, as well as motor signals, are represented in the cerebellum in a region-specific manner. Here we review recent findings on the circuit organization that underlies the optic flow processing driving OKR and OMR.

## Introduction

When an animal moves in an environment, either actively or passively, its displacement in the space causes its visual field to shift. Thus, an animal’s visual system is constantly activated by flow-like movements of the visual scene that are caused by its own movement (i.e., self-motion). Detecting such visual information, which is known as whole-field motion or optic flow, is essential for many animals because it serves as a feedback signal that allows them to estimate their own movement relative to the surrounding environment. In turn, the sensation of optic flow induces highly stereotyped behavioral responses of the eyes and body, by which animals adjust and compensate the displacement caused by the self-motion. These visuomotor behaviors are conserved across vertebrates, including teleost fish (Huang, 2008; Masseck and Hoffmann, 2009).

Over the past decade, significant progress has been made on our understanding of the neural circuits underlying optic flow processing in the larval zebrafish brain. In zebrafish larvae, functional imaging, mainly by means of calcium imaging, enables one to non-invasively and systematically probe response properties of neurons at a single cell resolution over a wide extent of the brain or even across the entire brain (Ahrens et al., 2012, 2013). Thus, this technological advance, combined with rich genetic techniques, in the larval zebrafish system provides an unparalleled opportunity to exhaustively identify neurons that respond to optic flow as well as reveal network functions by which optic flow inputs are converted to behavioral outputs.

In this review, we discuss recent discoveries of the neural circuits underlying optic flow processing in the larval zebrafish system. We will review the main cell types and brain regions, specifically retinal ganglion cells (RGCs), pretectum, and cerebellum, that process optic flow information and mediate the transformation of visual information to behavior.

### Optic Flow-Induced Behavior

An animal’s self-motion generates a drifting movement of the entire visual field called optic flow. Similar to other vertebrates, optic flow in zebrafish typically drives two behavioral responses, namely, the optokinetic response (OKR) and optomotor response (OMR). The OKR consists of two alternating phases that involve a rotating eye movement that tracks the perceived motion (slow phase), followed by a fast saccadic eye movement that flips the eyes back to the opposite direction (fast phase). The OMR is a swimming response in which the zebrafish swims in the direction of the optic flow. As a result, the directional motor behavior of the OKR and OMR serves to stabilize the gaze and body posture and/or position, respectively. Because the OKR and OMR are innate, robust behavioral responses that can be quantitatively measured already at larval stages (Clark, 1981; Easter and Nicola, 1997; Orger et al., 2000; Rinner et al., 2005; Portugues and Engert, 2009), they have served as instrumental assays for testing visual functions in forward genetic screens (Brockerhoff et al., 1995; Neuhauss et al., 1999; Muto et al., 2005). Horizontally moving stimuli and resulting horizontal OKR are used in most studies, but a torsional OKR induced by pitch motion has also been reported in larval zebrafish (Bianco et al., 2012).

Typically, OKR and OMR assays use simple, synthetic visual stimuli, such as sinusoidal or square gratings presented over a large field of view. What attributes of visual motion are actually extracted by the zebrafish visual system to induce OKR and/or OMR? The visual system detects motion by analyzing spatiotemporal patterns of light. First-order motion is defined by changes in luminance in space and time, whereas second- and higher-order motions are not defined by luminance modulations but rather by modulations of higher-order features, such as local contrast, flicker, or local motion. Zebrafish larvae perform OMR and OKR in response not only to first-order motion but also to second-order motion (Orger et al., 2000; Roeser and Baier, 2003). Furthermore, motion defined by three-point (third-order) correlations in space and time is also sufficient to effectively induce OMR in zebrafish (Yildizoglu et al., 2020) as well as flies (Clark et al., 2014). Additionally, by correlating components of visual features consisting of the forward-moving gratings and the elicited forward OMR bouts of the fish, it was found that not only the global whole-field motion, as expected from previous studies, but also a local spatiotemporal change of the luminance from light to dark close to the fish’s head were identified as key visual cues for OMR, indicating that the two features (i.e., global whole-field motion and local light–dark transition) work together to elicit the forward OMR (Kist and Portugues, 2019). In terms of color sensitivity, OMR is dominantly driven by red and green stimuli with a minimum contribution from UV/blue spectrum inputs (Orger and Baier, 2005).

### Optic Flow Processing Circuit: Linking Visual Inputs to Behavioral Outputs

**FIGURE 1:**
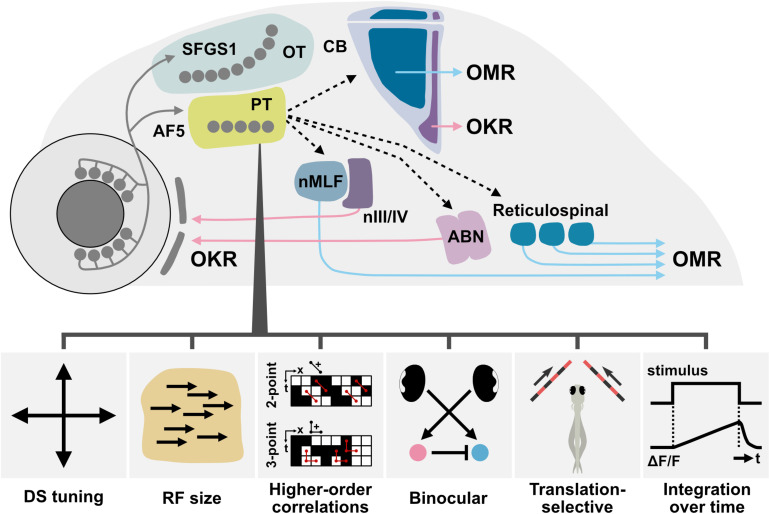
Organization of the optic flow processing circuit in the larval zebrafish brain. The direction selective (DS) retinal ganglion cells (RGCs) project to SFGS1 layer of the optic tectum (OT) neuropil and AF5 in the pretectal (PT) neuropil. DS neurons in the pretectum exhibit various response properties, such as four orthogonally arranged preferred directions, tuning for receptive field (RF) size and location, sensitivity to motion defined by higher-order correlations, binocular integration, translation-selective response, and temporal integration (see section “Pretectum” for details). For triggering OKR, the pretectum sends a signal (either directly or indirectly) to the oculomotor system [the oculomotor (nIII), trochlear (nIV), and abducens (ABN) nuclei] that contains motor neurons controlling the extraocular muscles. OMR swimming is regulated by the midbrain nucleus of medial longitudinal fasciculus (nMLF) and hindbrain neurons including the reticulospinal (RS) neurons, which likely receive visual inputs from the pretectum. nMLF and RS neurons are directly involved in controlling swimming of the fish via their descending axons reaching the spinal cord. In addition, rotation- and translation-selective information is represented in the rostromedial and caudolateral regions in the cerebellum (CB), respectively. OKR, optokinetic response; OMR, optomotor response. Solid lines indicate projections that have been shown in zebrafish larvae, whereas dotted lines represent proposed connections.

In this section, we briefly describe an overview of the optic flow processing circuit based on findings identified in zebrafish as well as other fish species (Figure 1). For both OKR and OMR, optic flow information in the visual scene is detected by the retina and transmitted to visual brain areas, mainly to the pretectum. For the OKR, the pretectum sends a signal (either directly or indirectly) to the oculomotor system that contains the motor neurons controlling the extraocular muscles (Masseck and Hoffmann, 2009). OMR swimming is regulated by the midbrain nucleus of the medial longitudinal fasciculus (nMLF) and hindbrain neurons including the reticulospinal (RS) system, which receive visual inputs from the pretectum. The nMLF and RS neurons are directly involved in controlling swimming of the fish via their descending axons, which reach the spinal cord (Orger et al., 2008; Severi et al., 2014; Thiele et al., 2014). In this mini review, we focus on three cell type/brain regions for which the functional and anatomical underpinnings of optic flow processing were recently uncovered, namely, retinal ganglion cells (RGCs), pretectum, and cerebellum.

### RGCs

RGCs are the output neurons of the retina that project their axons to the brain, thereby transmitting visual information to the brain. A subset of RGCs encodes a direction of visual motion, making them direction-selective (DS) (Barlow and Hill, 1963; Dhande and Huberman, 2014). In zebrafish larvae, RGC axons arborize in 10 anatomically distinct regions, termed arborization fields (AFs) and identified as AF1 to AF10 (Burrill and Easter, 1994; Robles et al., 2014), with the largest one being the neuropil of the optic tectum (AF10). *In vivo* calcium imaging of RGC axons that innervate the tectal neuropil/AF10 identified DS RGCs in zebrafish (Nikolaou et al., 2012). DS RGC axons are composed of three subtypes, each of which is tuned to a different direction of motion that is approximately 120° apart. These DS RGC axons are located only in the most superficial layer of the stratum fibrosum et griseum superficiale (SFGS) of the tectal neuropil. Furthermore, *in vivo* calcium imaging of RGC axon terminals in extratectal AFs identified DS RGC inputs terminating in AF5 and also partially in AF6 (Kramer et al., 2019). These AF5-targeted DS RGCs respond not only to a conventional grating motion but also to more complex motions, such as motion defined by three-point correlations, which can effectively induce OMR (Yildizoglu et al., 2020).

On the basis of the projection patterns of individual RGC axons, zebrafish RGCs are morphologically classified into 20 projection classes, each of which innervate a different combination of sublayers in the tectal neuropil/AF10 and extratectal AFs (Robles et al., 2014). One particular morphological class of RGCs projects to the SFGS1 sublayer of the optic tectum and also forms collateral branches in AF5 in the pretectal neuropil (Robles et al., 2014). Combined with the physiological evidence that DS RGC inputs are detected in SFGS1 (Gabriel et al., 2012; Nikolaou et al., 2012; Gebhardt et al., 2013) and AF5 (Kramer et al., 2019; Yildizoglu et al., 2020), this anatomical observation confirms that the SFGS1- and AF5-projecting class of RGCs corresponds to a DS RGC subpopulation. Thus, DS information is conveyed to both tectal and pretectal neurons via the same population of RGCs. However, it remains unknown whether the two postsynaptic targets of DS inputs (i.e., tectum/SFGS1 and pretectum/AF5) derived from the same DS RGCs are involved in different visual functions or behaviors. An ablation of RGC axons that innervate the tectal neuropil/AF10 (Roeser and Baier, 2003) and tectal neurons (Pérez-Schuster et al., 2016) showed that the optic tecum is not necessary for the generation of the OKR *per se*, but rather plays a role in the habituation of the OKR (Pérez-Schuster et al., 2016). Thus, it is possible that retinotectal DS inputs provide information required for the habituation of the OKR or they are involved in behaviors other than optic flow-induced responses, such as hunting of small moving objects during prey capture.

### Pretectum

The zebrafish pretectum is part of the diencephalon and is located ventrally to the optic tectum. Calcium imaging during whole-field motion revealed that DS neurons are highly enriched in the pretectum of zebrafish larvae (Kubo et al., 2014; Portugues et al., 2014; Naumann et al., 2016; Chen et al., 2018) and each DS neuron prefers one of four orthogonally arranged directions (Wang et al., 2019). Furthermore, optogenetic manipulation and laser ablation showed that the pretectum is essential for OKR (Kubo et al., 2014) and OMR (Naumann et al., 2016), suggesting that the pretectum is the principal brain area for processing optic flow in zebrafish and considered to be functionally homologous to the accessory optic system in mammals. The pretectum of zebrafish larvae consists of at least two functionally distinct regions: one is the optic flow-sensitive region described here, and the other corresponds to a more rostrally located region that is involved in prey capture behavior (Semmelhack et al., 2014; Muto et al., 2017; Antinucci et al., 2019).

The role of the pretectum in OKR and OMR predicts that pretectal neurons sample motion signals from a wide area of the visual field and/or local light-intensity transitions, as opposed to tectal neurons whose tuning to small-size moving stimuli agrees with their role in hunting small prey objects (Niell and Smith, 2005). Indeed, pretectal neurons have relatively large receptive fields (RFs) whose RF centers are located in the lower half of the visual field of the fish (Wang et al., 2020). In contrast, neurons in the tectum, which is dispensable for OKR and OMR (Roeser and Baier, 2003) and involved in detecting small objects for behaviors such as prey capture (Gahtan et al., 2005), have smaller RFs whose RF centers are located in the upper-nasal part of the visual field (Wang et al., 2020). Consistently, the presentation of forward translational motion in the lower visual field induces OMR more effectively than that in the upper visual field (Wang et al., 2020). On the other hand, the OKR is efficiently evoked by moving stimuli located laterally and near the equator of the fish’s visual field (Dehmelt et al., 2020). Consistent with their large RF size, pretectal neurons can integrate motion signals over space in a random dot motion kinematogram paradigm (Bahl and Engert, 2020; Dragomir et al., 2020). Interestingly, some pretectal neurons can also accumulate motion signals over time, suggesting that they act as a temporal integrator (Dragomir et al., 2020).

**FIGURE 2:**
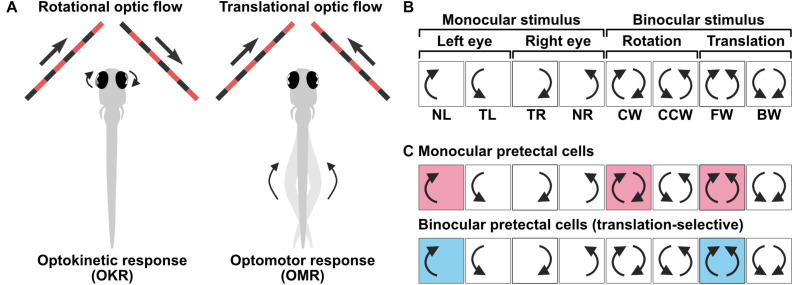
Representation of binocular optic flow information by pretectal neurons. **(A)** Rotational and translational optic flow trigger optokinetic response (OKR) and optomotor response (OMR), respectively. **(B)** 8-phase visual stimulus protocol used to characterize monocular and binocular selectivity of pretectal neurons. NL, nasalward motion to left eye; TL, temporalward motion to left eye; TR, temporalward motion to right eye; NR, nasalward motion to right eye; CW, clockwise; CCW, counter-clockwise; FW, forward; BW, backward. **(C)** (Top) Monocular direction selective pretectal cells respond to motion that moves in one direction (either nasal or temporal) presented to one eye. This example cell responds whenever a nasal motion is presented to the left eye, irrespective of the motion presented to the right eye. (Bottom) Translation-selective cells that responds to forward translational motion but no to rotational motion. In contrast to the cell shown above, the response to clockwise motion is suppressed although its response is predicted from the activity of this cell responding to nasal motion in the left eye.

In lateral-eyed animals, including zebrafish, comparing the motion information between the left and right eyes is an efficient strategy to estimate optic flow patterns across a wide extent of the visual field. The two most common optic flow patterns are rotation and translation, which are thought to mainly trigger OKR and OMR, respectively (Figure 2A). To test whether pretectal cells differentially represent rotational and translational optic flow patterns, response properties of a population of pretectal cells were examined via calcium imaging using a visual stimulus sequence that consisted of different monocular and binocular optic flow patterns in the horizontal plane (Kubo et al., 2014). These optic flow patterns consisted of four eye-specific DS monocular motions and rotational [clockwise (CW) and counterclockwise (CCW)] and translational [forward (FW) and backward (BW)] binocular motions (Figure 2B). By classifying pretectal cells into one out of all possible combinations of binary activity patterns in response to the eight stimulus phases (i.e., 2^8^ = 256 types) or to a “barcode” (Figure 2B), two major types of pretectum neurons were identified (Kubo et al., 2014). “Simple” monocular pretectal cells consist of four populations of DS neurons, each of which encodes either a nasal or temporal direction of motion presented to either the left or right eye (Figure 2C, top) and insensitive to the motion received by the other eye. In contrast, “complex” binocular pretectal neurons respond selectively to translational FW or BW motion, but not to CW and CCW rotational motion, indicating that different optic flow patterns are already distinguished in these cells (Figure 2C, bottom). Such binocular pretectal neurons were also reported using a slightly different visual stimulus presentation (Naumann et al., 2016; Wang et al., 2019). Mechanistically, the suppressed activity specifically during rotation but not during translation suggests that this suppression is provided by an input from the eye opposite to the one that activates the cells, thus rendering them binocular. Because the optic chiasm is completely crossed in zebrafish, pretectal binocular integration needs intra-pretectal commissures connecting both hemispheres of the pretectum. Indeed, ablation of the posterior commissure, which is a prominent commissure in the pretectal region, abolishes binocular integration (Naumann et al., 2016), suggesting that monocular information is transferred by the posterior commissure within the pretectum.

In addition to the aforementioned horizontal motion, two other major motion types (i.e., vertical and pitch motions) have been tested using a panoramic visual arena, thereby enabling investigation of the three-dimensional binocular encoding of optic flow (Wang et al., 2019). Approximately one-third of motion-sensitive tectal and pretectal neurons were “simple” monocular DS cells that responded to one of the four orthogonally arranged directions in one eye, irrespective of the motion presented to the other eye. Another one-third of the population was preferentially active only when one specific combination of binocular motion was presented to the left and right eyes and did not respond well to any other combinations of binocular motion, suggesting that a large fraction of pretectal and tectal neurons show translation- or rotation-selective representations in all three different axes (Wang et al., 2019). Such binocular pretectal neurons were detected irrespective of the visual field of the fish to which the visual stimulus was presented (Naumann et al., 2016; Wang et al., 2019). Thus, it is proposed that these selective pretectal neurons unambiguously encode appropriate directionality of OKR and OMR behaviors already at the level of the pretectum and no further sensory processing is, in principle, needed in the downstream circuit.

Although optic flow-responsive pretectal cells, be they monocular or binocular, are intermingled in the same pretectal region in larval zebrafish (Kubo et al., 2014; Naumann et al., 2016), their neurite projection patterns are different (Kramer et al., 2019). Namely, morphological characterization of functionally defined optic flow-responsive cells using a technique named function-guided inducible morphological analysis (FuGIMA) revealed that monocular DS pretectal cells extend dendrites to AF5 where DS RGC axons terminate (Kramer et al., 2019). In contrast, dendrites of binocular DS cells extend to dorsal AF6 and do not overlap with the region where DS RGC axons terminate (Kramer et al., 2019). These observations suggested a circuit model in which DS information of DS RGCs transmitted to AF5 is first inherited by monocular DS cells and then integrated in binocular DS cells through AF6 (Kramer et al., 2019). Pretectal projection neurons identified using a single cell atlas of zebrafish brain (Kunst et al., 2019) project axons to the reticular formation, tegmentum, hypothalamus, and cerebellum, suggesting that these brain regions are candidates for receiving pretectal-derived optic flow information downstream of the pretectum.

Optic flow-responsive cells in the pretectum are roughly organized in spatial clusters (Kubo et al., 2014). One of the clusters located in the ventral–lateral region contains neurons that respond to a classical motion illusion known as motion aftereffect (MAE) (Wu et al., 2020), which refers to a perception of illusory motion after a continuous exposure to a moving stimulus in one direction (Pérez-Schuster et al., 2016; Lin et al., 2019). These cells in the ventral–lateral pretectal cluster, consisting of a small number of neurons (∼12 neurons per fish), are largely monocular DS (Wu et al., 2020). Ablation and optogenetic activation studies showed that these MAE-correlated DS neurons are essential for OKR, suggesting that this rather small population of DS neurons in the ventral–lateral pretectum is an integral part of the optic flow-responsive circuit.

In adult zebrafish, the pretectum is subdivided into several retinorecipient and non-retinorecipient nuclei based on cytoarchitecture and efferent and afferent pathways (Wullimann et al., 1996; Yáñez et al., 2018). The correspondence between adult pretectal nuclei and optic flow-responsive pretectal neurons in larvae remains unclear. However, a recent study comprehensively matching the function and anatomy between larvae and adults proposed that AF5-pretectal circuits in larvae correspond to the dorsal accessory optic nucleus (DAO) of the adult pretectum (Baier and Wullimann, 2021).

In summary, pretectal cells not only encode monocular optic flow signals (much like DS RGCs) but also respond to a much wider variety of optic flow features, such as binocularly integrated optic flow, and these response properties have already been tailored to compute behaviorally relevant information. Future work is required to elucidate the circuit mechanism and connectivity by which pretectal cells integrate optic flow information across space and time.

### Cerebellum

The cerebellum is known as a major brain region that controls motor coordination and learning (Ito, 2006). Several studies using zebrafish have demonstrated cerebellar activation during optic flow stimuli (Ahrens et al., 2012; Matsui et al., 2014; Portugues et al., 2014) as well as functional roles for the cerebellum in motor coordination, adaptation, and learning (Aizenberg and Schuman, 2011; Ahrens et al., 2012; Harmon et al., 2017; Matsuda et al., 2017).

Compared with the pretectum, cell type composition, cellular organization and connectivity are better characterized in the cerebellum. The larval zebrafish cerebellum is anatomically organized in a typical vertebrate trilayered structure, consisting of the two major cell types, gamma−aminobutyric acid (GABA)ergic Purkinje cells (PCs) and glutamatergic granule cells (GCs) (Bae et al., 2009; Hashimoto and Hibi, 2012; Hsieh et al., 2014; Hamling et al., 2015). PCs receive afferent inputs from climbing fibers that originate from the inferior olivary nuclei located in the caudal hindbrain. In contrast, GCs receive inputs from mossy fibers that originate from neurons in several precerebellar nuclei located in various brain regions. GC axons further convey the information to PCs through parallel fibers. PCs integrate the two sources of inputs and finally send their outputs outside the cerebellum, either directly or indirectly via eurydendroid cells, which are the sole output neurons of the cerebellum (equivalent to the deep cerebellar nuclei in mammals). Single cell reconstruction and tracer studies have identified neuronal connections from the pretectum to the cerebellum in larval (Kramer et al., 2019; Kunst et al., 2019) and adult (Yáñez et al., 2018; Dohaku et al., 2019) stages. However, it remains to be tested whether these pretectal–cerebellum projections carry optic flow-related signals, in other words, whether optic flow-responsive pretectal neurons directly project to the cerebellum.

Imaging of neuronal activity across the whole cerebellum population revealed regional differences in the cerebellum (Matsui et al., 2014; Knogler et al., 2019). In a pioneering work by Matsui et al. (2014), calcium imaging of PCs revealed that the rostromedial area of the cerebellum was activated during OMR, whereas the caudal part of the cerebellum was activated during OKR. These OMR- and OKR-related neuronal responses in PCs were absent when the tail or eyes of the fish were restrained, suggesting that these responses were related to proprioception and/or motor signals (Matsui et al., 2014). Furthermore, optogenetic manipulation of the rostromedial and caudal PC populations impairs tail movements triggered by OMR and eye movements induced by OKR stimulus, respectively (Matsui et al., 2014). Thus, cerebellar PCs are highly regionalized such that different functions are organized in rostromedial and caudal regions. Moreover, these functionally distinct regions have distinct afferent projection patterns. The rostromedial cerebellum projects to locomotor-related regions, such as nMLF, red nucleus, thalamus, and reticular formation, whereas the caudal cerebellum projects mainly to a vestibular-related region, namely, the descending octaval nucleus (Bae et al., 2009; Matsui et al., 2014; Knogler et al., 2019). Taken together, these functional and anatomical observations suggest that functionally distinct motor information is relayed to distinct downstream pathways, thereby driving the divergent motor outputs required for OKR and OMR.

Building on this finding, Knogler et al. (2019) examined whether PCs receive visual or motor inputs by presenting translational and rotational motions and simultaneously recording tail and eye movements. Taking advantage of the fact that variables encoding the visual inputs and behavioral outputs are correlated but temporally separable, the authors disambiguated whether PCs responded to either visual or motor variables (Knogler et al., 2019). Electrophysiological recordings of single PCs allowed the authors to separately analyze the two excitatory input streams that PCs receive, namely, complex spikes that originate from climbing fibers from the inferior olivary nucleus and simple spikes that arise from parallel fibers of GCs (Knogler et al., 2019). Inputs from the climbing fiber, which were measured by complex spikes, conveyed sensory, but not motor, information. Interestingly, climbing fiber inputs carrying translational motion information (i.e., OMR-triggering visual information) were frequently represented in the rostromedial region, whereas those carrying rotational motion information (i.e., OKR-triggering visual information) were highly abundant in the caudolateral region. In contrast, inputs from GC-derived parallel fiber measured by simple spikes were highly correlated with motor activity of the fish (measured by ventral root recordings in fictive swimming preparations), since such motor-related simple spikes were observed even without visual stimulation (Sengupta and Thirumalai, 2015; Scalise et al., 2016; Knogler et al., 2019). Consistent with these motor-related properties of simple spikes, GCs themselves were also motor correlated (Knogler et al., 2017, 2019). In summary, the cerebellum is spatially organized into behavioral modules, in which the two input streams (i.e., inferior olive-derived sensory stream and GC-derived motor stream) converge and integrate in PCs in a region-specific manner, and thus represents distinct visual features with motor context.

One of the major hypotheses for the role of the climbing fibers and complex spikes in the cerebellum is that climbing fiber input conveys error signals, i.e., discrepancies between a motor command and a feedback signal of the produced motor outcome, such as an unexpected image motion or retinal slip (Ito, 2013; Streng et al., 2018). Such error signals play a teacher’s role for correcting subsequent behavior. When larval zebrafish passively experience optic flow and receive no visual feedback upon their own movements, an error signal is likely to be generated. However, evidence so far has not definitively identified the encoding of error signals in the cerebellum of larval zebrafish (Scalise et al., 2016; Knogler et al., 2019). Since the error hypothesis has been developed mostly in the context of learning, it is possible that different principles apply for the innate coding of sensory features during OKR and OMR in naïve animals. Thus, it remains to be concluded what exact signals are carried by climbing fibers in the larval cerebellum (e.g., error/novelty/salience).

## Outlook

As discussed in this mini review, a series of recent studies uncovered the general organization of the optic flow processing pathway, involving a dedicated channel for DS processing in RGCs, integration of sensory information in the pretectum, and sensorimotor transformation and regionalization in the cerebellar circuit (Figure 1). Most of these discoveries were made possible by functional imaging at the systematic and cellular level as well as by testing a wider parameter space of visual stimulations. Although functional imaging has exhaustively identified key brain regions and cell populations for optic flow processing, this approach can, by definition, only correlate neuronal activity with sensory or motor variables, but cannot prove connectivity of neurons within or between given brain areas. To overcome this limitation and go beyond correlational analysis, other analysis approaches, such as spatiotemporally specific functional manipulations and anatomical or molecular analyses, will be required to cohesively understand the whole network mechanism that mediates optic flow processing and behavior.

## Author Contributions

KM and FK drafted the manuscript. Both authors contributed to the article and approved the submitted version.

## Conflict of Interest

The authors declare that the research was conducted in the absence of any commercial or financial relationships that could be construed as a potential conflict of interest.
